# Women’s Respiratory Movements during Spontaneous Breathing and Physical Fitness: A Cross-Sectional, Correlational Study

**DOI:** 10.3390/ijerph191912007

**Published:** 2022-09-22

**Authors:** Wen-Ming Liang, Zhen-Min Bai, Maiwulamu Aihemaiti, Lei Yuan, Zhi-Min Hong, Jing Xiao, Fei-Fei Ren, Osvaldas Rukšėnas

**Affiliations:** 1 Life Sciences Center, Vilnius University, LT-10257 Vilnius, Lithuania; 2 Department of Physiotherapy and Rehabilitation, Xiyuan Hospital, Chinese Academy of Chinese Medical Sciences, Beijing 100091, China; 3 School of Sports Medicine and Rehabilitation, Beijing Sport University, Beijing 100084, China; 4 School of Science, Inner Mongolia University of Technology, Hohhot 010051, China; 5 Department of Physical Education, Beijing Language and Culture University, Beijing 100083, China

**Keywords:** abdominal motion, thoracic motion, physical performance, countermovement jump height, cardiorespiratory endurance, body size

## Abstract

Background: Abdominal/diaphragmatic breathing exercises are popular worldwide and have been proven to be beneficial for physical performance. Is abdominal motion (AM) during spontaneous breathing correlated with physical fitness? The present study aimed to answer this question. Methods: 434 women (aged 20–59) were enrolled and participated in respiration tests using two respiration belts (one was tied at the height of the xiphoid and another at the navel) to detect AM and thoracic motion (TM). They also performed physical fitness tests to measure body size, muscular strength, muscular power, muscular endurance, balance, flexibility, reaction time, and cardiorespiratory endurance. Results: All the correlation coefficients between respiratory movements (AM, TM, AM + TM, AM/(AM + TM)) and physical fitness outcomes were less than 0.4/−0.4. Only AM and muscular power (countermovement jump height) had a weak correlation, with a correlation coefficient close to 0.4 in the 20−29-year age group (r_s_ = 0.398, *p* = 0.011, n = 40). Conclusions: Women’s respiratory movements during spontaneous breathing were not correlated with physical fitness. Future studies may focus on the relationship between AM and countermovement jump height in young women with a larger sample size and using ultrasound to directly test the excursion of the diaphragm.

## 1. Introduction

Respiration is vital to our health, as it not only provides us with O_2_ and expels CO_2_, but is also important for vocalization, pH and temperature regulation, pathogen prevention, and gastrointestinal motility [1,2,3]. The function of respiration is closely related to breathing patterns since breathing pattern involves respiration rate, the ratio of inspiration to expiration, depth, chest wall movement, and the exertion of accessory respiratory muscles [4]. Emotional, chemical, and postural factors are able to alter breathing patterns [5,6]. Interactively, breathing patterns influence neural activity and core stability [7,8,9]. Each person has their own respiratory pattern. One might have a longer exhalation duration and larger chest movement, while another might inhale longer and have larger abdominal motion. In the present study, we focused on respiratory movements, specifically the motion of the chest and abdomen.

We can consciously control our respiratory movements through activation of the motor cortex, but most of the time, breathing is unconsciously activated by the respiratory center in the medulla oblongata [1]. Noteworthily, voluntary breathing training was found to be able to reform involuntary respiration movements [10]. There are plenty of voluntary-controlled breathing exercises; abdominal breathing, also called diaphragmatic breathing, is a popular type that is attracting more and more attention for health improvement [11].

Abdominal breathing is breathing that is dominated by the movement of the diaphragm that expands the abdominal wall if chest movement is controlled, while thoracic breathing is dominated by accessory inspiratory muscles (e.g., external intercostal muscles, scalenus, pectoralis, and sternocleidomastoid) that increase the diameter of the thoracic cavity [12,13]. Scientific research has found that abdominal breathing exercises are beneficial not only for mental health [14,15], but also for physical wellbeing (cited research as follows). Six-minute walking distance was significantly improved in COPD patients after abdominal breathing exercises, as concluded by a meta-analysis study [16]. Cavaggioni et al. found that core stabilization exercises combined with diaphragmatic breathing and global stretching postures improved pulmonary function and abdominal fitness more effectively in healthy males [17]. Barbosa et al. observed that using abdominal drawing-in breathing during squats increased activation of the rectus, biceps femoris, and tibialis, and increased movement stability [18]. Martarelli et al. found that abdominal breathing could reduce exercise-induced oxidative stress, which was supposed to protect athletes from the long-term adverse effects of free radicals [19]. Remarkably, Nelson summarized that abdominal breathing enhanced core and trunk stability and addressed that better breathing habits should positively affect core stability and ultimately improve the overall conditioning of the athlete [20]. Following Nelson’s theory, studies regarding the relationship between involuntary respiration movements and physical fitness were reviewed. Teixeira-Salmela et al. observed that community-dwelling people with chronic stroke had less abdominal contributions to spontaneous breathing [21]. Diaphragmatic mobility and respiratory muscle endurance were lower in patients with non-specific low back pain [22]. Kocjan et al. found that larger diaphragm motion during spontaneous breathing is associated with better balance stability in patients after lung resection due to cancer and in healthy participants [23]. According to the above context, less abdominal contributions to spontaneous breathing were observed in patients with stroke and lower back pain, while large diaphragm motion during normal quiet breathing was correlated with better balance performance in patients and healthy participants.

Theoretically, the work of the diaphragm has an influence on physical performance. The diaphragm is one of the critical core muscles for trunk stability [24], as the excursion of the diaphragm controls intra-abdominal pressure and reduces the stress on the spine through cooperation with the abdominal and pelvic floor muscles, and the stability of the trunk is the basis of all functional movements [25,26,27]. Furthermore, the contraction of the diaphragm is associated with respiratory movements. Indeed, Talasz et al. studied the phase-locked parallel movement of the diaphragm, pelvic floor muscles, and abdominal wall during quiet and forced breathing in healthy women, and demonstrated increased exertion of the diaphragm, and greater motion of the pelvic floor muscles and the abdominal wall during inspiration, and vice versa during expiration [28]. This means abdominal motion (AM) could demonstrate the mobility of the diaphragm and pelvic floor muscles, which work together for core stability. Through reviewing the literature, we noticed that studies related to the work of the diaphragm/respiratory movements during spontaneous breathing and physical fitness in a healthy population are rare. Some studies found that respiratory muscles were involved in various physical activities in which respiration was not primarily involved. For example, the diaphragm was recruited during weight-lifting [29]; the transdiaphragmatic pressure increased as the arm flexed rapidly [30], and diaphragm EMG amplitude correlated with the peak upper limb acceleration when participants moved with increasing frequency [31]. The study conducted by Kocjan et al. demonstrates that diaphragm motion during spontaneous breathing and balance stability was positively correlated [23]. However, the study only tested balance performance. A study covering more physical fitness components would give a more comprehensive understanding of the relationship between respiratory movements and physical health.

Physical fitness is a fundamental indicator of a healthy state [32], and extensive tests for physical fitness can present one’s physical well-being in a comprehensive way. Physical fitness comprises health-related fitness and skill-related fitness. Health-related fitness includes muscular strength, muscular endurance, flexibility, cardiorespiratory endurance, and body composition; skill-related fitness includes speed, power, agility, balance, coordination, and reaction time [33]. Thus, the present study aimed to investigate the relationship between spontaneous respiratory movements and physical fitness components.

Participants were enrolled through convenience sampling. We planned to investigate both men and women. However, 71% of the participants that enrolled were women. To eliminate the effect of age and to detect the specific age range in which respiratory movements might correlate with physical fitness, we planned to divide participants into four age groups for statistical analysis. Because of this, the sample size was too small for the men’s data. Therefore, to ensure the reliability of our results, we focused on the women’s data and hypothesized that healthy women’s abdominal motion during spontaneous breathing would be positively correlated with physical fitness (except body size). These findings might present a new aspect to consider for physical health improvement.

## 2. Materials and Methods

### 2.1. Trial Design and Participants

This is a cross-sectional correlational study. A total of 434 healthy females (aged 20–59) were enrolled through convenience sampling from six communities in Haidian District in Beijing, and they were divided into four age groups at ten-year intervals for statistical analysis. Inclusion criteria were as follows: participants who (1) were aged 20–59 years; (2) were capable of understanding and answering the interview questions; (3) filled out the Physical Activity Readiness Questionnaire and met all the requirements; (4) provided written informed consent. Exclusion criteria were as follows: participants who (1) were pregnant or lactating women; (2) suffered from a mental illness; (3) suffered from acute diseases or had suffered from acute diseases and had not recovered physically; (4) drank coffee or tea 2 h before the tests; (5) had a respiration rate exceeding the average ± 2 times standard deviations in their age group (excluded subject: RR < mean – 2 × SD, or RR > mean + 2 × SD); (6) did not perform ten consecutively stable respiratory cycles from a two-minute respiration test.

The study was approved by the Research Ethics Committee of Beijing Sport University (Approval number: 2021079H), and all subjects were informed of the risks of the tests prior to signing the informed consent document.

### 2.2. Measures

In the present study, participants performed tests for respiratory movement (abdominal motion and thoracic motion), body size (height, weight, waist circumference, and hip circumference), body composition (body fat percentage), muscular strength (handgrip strength and back extension strength), muscular power (countermovement jump height), muscular endurance (number of push-ups and sit-ups), balance (one-leg stance test), flexibility (sit and reach test), reaction time (simple visual reaction time test), and cardiorespiratory endurance (YMCA submaximal cycle ergometer test for VO_2_max).

#### 2.2.1. Respiratory Movements Testing and Data Processing

**Figure 1:**
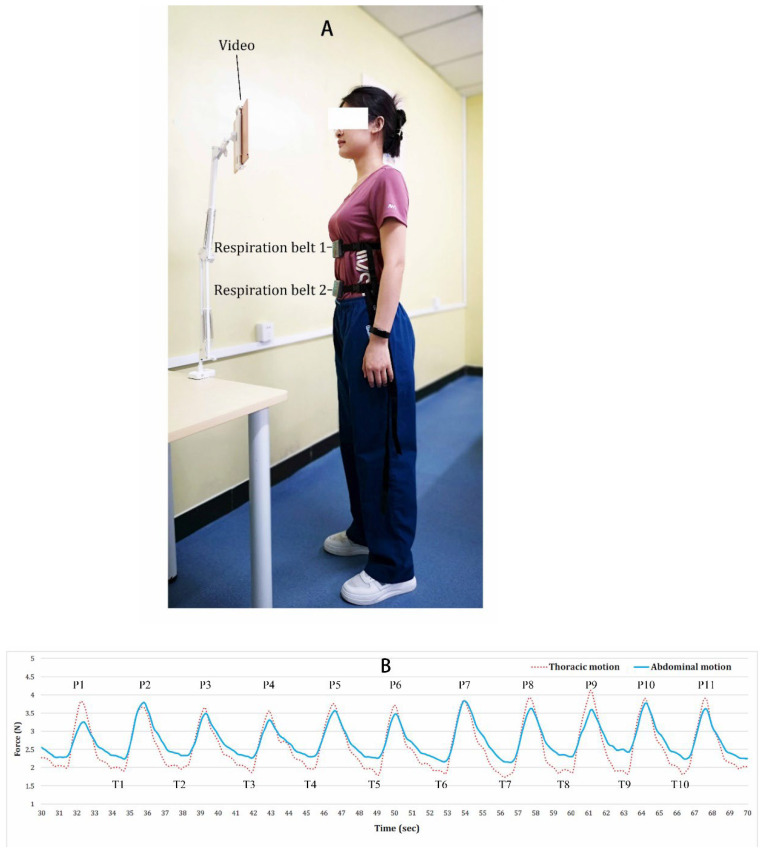
(**A**) Respiration testing. Respiration belt 1 was fastened at the height of the xiphoid; respiration belt 2 was fastened at the height of the navel; a neutral video was playing. (**B**) Wave lines of respiration. The force was generated by the stretch of respiratory movement; solid line represents the abdominal motion; dotted line represents the thoracic motion. P = peak; T = trough.

The testing of respiratory movements was conducted using respiration belts (Vernier, Beaverton, OR, USA), which is a strap of fabric with a resistive stretch sensor embedded into it. Researchers used one or two belts to test the respiration rate [34], breathing maneuvers (abdominal breathing and chest breathing) [35], or respiratory waveform [36]. In the present study, we used two belts. Before testing, participants were asked to sit quietly for 5 min to calm down mentally and physically. Then, they stood up to eliminate the influence of abdominal circumference. One belt was tied at the height of the xiphoid and another at the navel for detecting the movement of the chest and abdomen (Figure 1A). The research tightened the strap on the subject until the light on the belt turned green or loosened the strap if the light turned red, according to the user instructions. During the test, subjects were asked to watch a neutral video to distract their attention from breathing. One Xiaomi Pad (size: 11 inches, resolution: 2560 × 1600; Xiaomi, Beijing, China) displayed the video in front of the subject’s face at a distance of 50–80 cm. The content of the video was small fishes swimming slowly in the sea.

The two belts were operated simultaneously, and the test lasted two minutes. Different authors used different methods to choose the number of respiratory cycles for analysis—from three satisfactory readings to six minutes of breathing cycle [21,23,37]. We observed that generally the respiration waves became stable after 30 s from the start. Therefore, we selected ten consecutively stable respiration cycles with minimal motion artifact and baseline wander after 30 s from the start of the testing. Data were imported from the Vernier Graphical Analysis (Vernier, Beaverton, OR, USA) to OriginPro 9.0 (OriginLab, Northampton, MA, USA) for extracting peaks and troughs of breathing waves. After, the peaks and troughs were imported to Excel (Excel, Microsoft, Redmond, USA) for calculating abdominal motion (AM), thoracic motion (TM), and respiration rate (RR). AM and TM were calculated separately, and the values of AM and TM were determined as the ten averaged peak (P) forces minus ten average trough (T) forces (motion = (P1 + P2… + P10)/10 − (T1 + T2… + T10)/10), while respiration rate was determined as 60 s divided by the time used for one respiration cycle, which was calculated from the time of the 11th peak minus the time of the 1st peak divided by 10 (RR = 60/(P11 − P1)/10) (Figure 1B). The signals were presented as force (unit = Newton) with a sampling frequency of 10 Hz.

#### 2.2.2. Physical Fitness Testing

All physical fitness tests were conducted on an electronic physical fitness assessment system (Jianmin, Xindonghuateng Sports Equipment Co., Ltd., Beijing, China). Every participant had a card that each testing equipment could sense, and all testing results were stored directly in this system. The system was approved by the Sports Equipment Approval Committee at the General Administration of Sport, and the protocol of measurements was made and implemented according to the book National Physical Fitness Testing and Evaluation [38].

Body height measurement: Participants had their body height measured with shoes off using an electronic body height measuring instrument (Jianmin GMCS-SGJ3, Xindonghuateng, Beijing, China). As most of the equipment for the physical fitness tests were made by this company, we will state the brand “Jianmin” and the model, hereafter. Accuracy was to 1 cm.

Body weight measurement: Participants had their body weight measured wearing light clothes with shoes off on an electronic weighing scale (Jianmin GMCS-RCS3). Accuracy was to 0.1 kg.

Body fat test: The body fat test was performed on a body composition analyzer (Jian min GMCS-TZL3) with bare feet standing on two electrode plates and hands holding two electrode handles for one minute. Accuracy was to 0.1 kg.

Waist and hip circumference measurement: Waist and hip circumference was measured by an electronic circumference measuring ruler (Jianmin GMCS-WD3) at the height of the navel and the widest part of the buttocks. The accuracy was to 0.1 cm.

**Figure 2:**
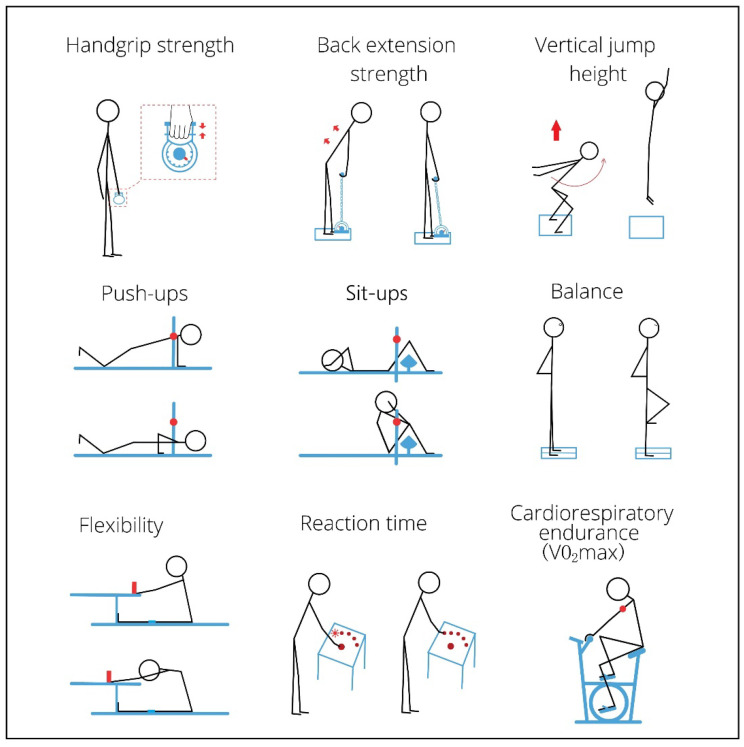
An illustration of the tests of physical fitness.

Muscular strength tests: Prior to the strength test, subjects were informed about all testing procedures and were recommended to engage in a 5 min warm-up. Then, participants stood and held a handgrip dynamometer (Jianmin GMCS-WCS3) in the dominant hand, 10–20 cm away from the thigh with the palm facing toward the thigh. Two minutes after the handgrip strength test, subjects participated in a back extension test with another dynamometer (Jianmin GMCS-BLJ3) that consisted of a plate for standing on, a bar for hand holding, and a chain connecting the plate and bar. The subject stood on the plate with hands dropped down and fingers straightened in front of two thighs. Then, the researcher adjusted the length of the chain and set the bar at the height of the tips of the middle fingers of subjects. Subjects flexed their hips and held the bar (keeping the arms, legs and trunk straight), and then slowly lifted up the bar with as much power as they could (Figure 2). The muscular strength tests were performed twice, and the best result was recorded with an accuracy of 0.1 kg.

Muscular power test: The countermovement jump with arm swing was adopted to test vertical jump height. Participants stood on a timing mat (Jianmin GMCS-ZTJ3) that contained an internal calculator for recording the individual’s time in the air and computing how high their jump was (Figure 2). Participants jumped two times, and the highest value was recorded with an accuracy of 0.1 cm.

Muscular endurance tests: Participants faced the floor with their weight distributed to the hands (straight) and knees (bent for 90°) on push-up counting equipment (Jianmin GMCS-FWC3), and one assistant adjusted two laser detectors to the height of participants’ shoulders. Then, participants performed the common push-up method for one minute. The system recorded when participants pushed their trunks up and their shoulders reached that height. The number of sit-ups performed in one minute was recorded in a similar way with sit-ups counting equipment (Jianmin GMCS-YWQZ3), as shown in Figure 2.

Balance test: Participants stood with arms akimbo in two spots on balance testing equipment (Jianmin GMCS-DJZL3). Once they were ready, they closed their eyes and lifted one foot. The system started recording the time when the participant lifted their foot and stopped the recording when another foot moved away (Figure 2). The accuracy of standing time was 0.1 s.

Flexibility test: Participants took off their shoes and sat on the equipment (Jianmin GMCS-TQQ3) with legs stretched out against one box, and the assistant fastened their knees to the equipment to keep their knees straight. With their palms facing downwards, participants reached forward along the measuring bar as far as possible (Figure 2). The test was performed twice, and the longer distance was recorded with an accuracy of 0.1 cm.

Reaction time test: The panel for testing reaction time (Jianmin GMCS-FYS3) has one starting button and five signal buttons. Participants stood in front of the panel and kept their dominant hand on the starting button. One of the signal buttons lit up randomly during each trial, and participants pushed the lit-up signal button as fast as possible. Five trials were performed for each round, and the average time of the five trials was used as the result. Participants participated in two rounds and the shorter time was recorded with an accuracy of 1 millisecond (ms).

Cardiorespiratory endurance test: The maximal oxygen consumption (VO_2_max) was estimated using a YMCA submaximal cycle ergometer [39]. After a five-minute rest, participants sat on the cycle ergometer (Jianmin GMCS-GLC3) with an optical heart rate sensor (Polar OH1, Polar Electro Oy, Kempele, Finland) worn around the upper arm. The sensor was connected to the physical fitness assessment system via Bluetooth, so the system could calculate the heart rate and add workloads. The test lasted seven minutes with 30 s for the baseline heart rate, three minutes for the first stage heart rate, another three minutes for the second stage heart rate, and the final 30 s for cooling down. A detailed calculation method can be found in the study conducted by Nuria Garatachea et al. [40].

### 2.3. Normalizing the Outcomes of Tested Physical Parameters

Body size is generally believed to be a confounding factor in the outcomes of physical performance tests [41,42]. Absolute values from physical tests might introduce bias to existing muscle strength for clinical reference. For instance, handgrip strength/body weight was a better clinical predictor of functional impairments than absolute value [43]; muscle strength, normalized for body weight, height, and fat mass is superior to absolute muscle strength as a predictor of cardiometabolic risk [44]. Thus, normalizing physical performance for body size is necessary. The most often employed equation was:

Pn = P/Mb(1)

where P is the physical performance; Pn is the normalized physical performance; M is the body mass; and b is the allometric value [45,46]. Markovic and Jaric proposed three allometric values for three types of physical performance: b = 0.67 for the tests of exertion of external force (e.g., hand grip strength), b = 0 for the tests of rapid movements (i.e., jump height), and b = −0.33 for the tests of supporting body weight (push-ups, pull-ups, maintaining strength demanding postures in gymnastics or yoga) [46].

The present study normalized handgrip strength and back extensor strength using 0.67 as the allometric parameter. Vertical jump height was found to be a body size-independent index [47,48], which was not likely to require normalization for body size [45]. Therefore, the original jump height was used. Results for push-ups, sit-ups, and single-leg stance duration (balance) were negatively correlated with body weight, so the allometric parameter (−0.33) was employed in Equation (1). Flexibility and visual reaction time were not significantly correlated with body weight. These two physical outcomes could be regarded as body size-independent; thus, the original values were used. For VO_2_max, the components for its calculation include age and body weight, which means it has already been normalized, and no further normalization is needed. Thus, the original VO_2_max values were used.

### 2.4. Controlling for the Influence of Age

Muscle strength is substantially affected by aging [49]. In the present study, we divided participants into four age groups for two purposes: to eliminate the effect of age and to detect the specific age range in which respiratory movements might be associated with physical performance. The first purpose was accomplished to a certain extent. Nevertheless, age is still significantly correlated with three sets of data: jump height in the 40s and 50s age groups (r_s_ = −0.170, *p* < 0.05; r_s_ = −0.233, *p* < 0.05) and the number of sit-ups in the 50s age group (r_s_ = −0.265, *p* < 0.05). To further eliminate the influence of age, we generated three sets of residuals that combined age with each set of data (from the three sets mentioned above) using linear regression on SPSS 20.0 (IBM, New York, NY, USA). We used the generated residuals to conduct a correlation test with respiratory movements during statistical analysis. This method (using residuals) is exactly the same as the semipartial correlation processes that eliminate one confounding factor’s effect [50].

### 2.5. Statistical Analyses

The Shapiro–Wilk test was performed to assess the data’s distribution. Linear regression was used to generate residuals for certain physical outcomes that were significantly influenced by age. Curve estimation regression (quadratic and linear models) was used to test the linear or non-linear relation. Spearman’s correlation works for non-parametric data and data that follow curvilinear relationships [51,52]. The data on respiratory movements did not meet the normality requirement, and the quadratic model of curve estimation fitted better for the relationship between respiratory movements and physical fitness outcomes. Therefore, Spearman’s correlation test was used to assess the correlation between respiratory movements and physical fitness outcomes. Significance was determined at an alpha level of *p* < 0.05. We accepted the interpretation of the strength of correlation coefficients according to Prion and Haerling’s study, as shown in Table 1 [53]. A correlation coefficient greater than 0.4 was set as the minimum correlation coefficient to accept the hypothesis. All statistical analysis was performed using SPSS 20.0.

## 3. Results

A total of 434 healthy women participated in respiratory and physical tests. Sixteen of them were excluded because of irregular respiratory waves, where we could not obtain ten consecutively stable respiratory cycles. Another sixteen were excluded due to respiration rate (nine participants’ respiration rates were too high, and the other seven participants’ rates were too low), and one participant’s respiration data were missing. Consequently, 401 subjects were included and divided into four age groups at ten-year intervals for analysis.

The basic characteristics of participants and outcomes of physical fitness were presented as median, first quartile, and third quartile, as the distribution of the data from respiratory movements and some physical fitness outcomes (e.g., balance, number of sit-ups and push-ups) was skewed (Table 2).

The correlations between respiratory movements and age, body height, body weight, BMI, waist circumference, hip circumference, waist circumference to hip circumference, and body fat percentage were insignificant (Table 3).

The relationships between respiratory movements and physical fitness were depicted in Table 4. All correlations between respiratory movements and physical fitness were less than 0.4/−0.4. Only abdominal motion (AM) and countermovement jump height had a weak correlation, with a correlation coefficient close to 0.4 in the 20s age group (r_s_ = 0.398, *p* = 0.011). Thoracic motion (TM) was significantly correlated with the number of sit-ups in the 30s age group (r_s_ = 0.238, *p* = 0.016), which was the only significant correlation found from TM. Abdominal motion plus thoracic motion (AM + TM) was significantly correlated with the number of sit-ups in the 30s age group (r_s_ = 0.330, *p* = 0.001), with the number of push-ups in the 50s age group (r_s_ = 0.211, *p* = 0.044), and with vertical jump height in the 30s and 40s age groups (r_s_ = 0.211, *p* = 0.025; r_s_ = 0.187, *p* = 0.024). The correlation between AM + TM and physical fitness was not consistent in different age groups and different physical performances. The only significant correlation between the proportion of AM to AM plus TM (AM/(AM + TM)) and physical fitness outcomes was with vertical jump height in the 40s age group (r_s_ = 0.178, *p* = 0.032).

## 4. Discussion

This study was a cross-sectional study that aimed to investigate the relationship between respiratory movements and physical fitness. A total of 434 healthy women from urban areas were enrolled. All the physical fitness components were tested using one electric physical fitness assessment system. We normalized physical performance and body size and controlled the influence of age. These measures were carried out to eliminate the influence of confounding factors and increase the results’ reliability.

The main finding in the present study was that all correlation coefficients among respiratory movements and physical fitness outcomes were less than 0.4/−0.4. Therefore, our hypothesis could not be confirmed. Unexpectedly, this study confirmed that respiratory movements were not correlated with physical fitness in females, especially for the population aged 40–59, as all correlation coefficients from their tests were less than 0.211.

Nevertheless, we noticed that abdominal motion (AM) and countermovement jump (CMJ) height had a weak correlation, with a correlation coefficient close to 0.4 in the 20s age group (r_s_ = 0.398, *p* = 0.011). We suggest that CMJ (with arm swing) requires lower limb explosive power [54,55], as well as refined muscular coordination, as it requires the activation of stretch reflex (or myotatic reflex, muscle stretch-shortening cycle) on the legs and arms [56,57,58,59]. Therefore, participants need a well-functioning trunk to integrate movement of the legs and arms to generate more force for jumping. As mentioned in the introduction, the stability of the trunk is the basis of all functional movements [25]. The diaphragm is one of the critical core muscles for trunk stability [26], as it works to control intra-abdominal pressure and reduce stress on the spine through cooperation with the abdominal and pelvic floor muscles [27]. To summarize, AM has the potential to influence CMJ. We suggest conducting further studies to verify this relationship in young women using ultrasound, since ultrasound can directly test the amplitude of excursion of the diaphragm.

With respect to thoracic motion (TM), it correlated significantly only with sit-ups in the 30s age group (r_s_ = 0.238, *p* = 0.016). Except for this weak correlation, the association between TM and other physical outcomes was inconsistent and very weak. According to the values of AM/(AM + TM) presented in Table 2, TM was dominant in breathing movement, which is in line with previous studies where women had a greater thoracic contribution than abdominal contribution during quiet breathing [60,61]. In addition, a healthy adult woman’s tidal volume is approximately 46 mL [62]. This means that TM is basic to quiet breathing; a healthy woman does not need much force to conduct it. Therefore, the magnitude of chest movement is similar among healthy women. Consequently, TM does not correlate with physical fitness.

Abdominal motion plus thoracic motion (AM + TM) can be regarded as tidal volume since the movement of the diaphragm and supplementary respiratory muscles deform the abdomen and chest wall. Even though AM + TM was significantly correlated with the number of sit-ups in the 30s age group (r_s_ = 0.33), with vertical jump height in the 20s and 40s age group (r_s_ = 0.211, *p* = 0.187), and with the number of push-ups in the 50s age group (r_s_ = 0.211), the relationships were weak or negligible. Since no previous study was found regarding the association between tidal volume/AM + TM and physical fitness, these findings could be a reference point for further studies.

AM/(AM + TM) is the contribution of abdominal motion to abdominal motion plus thoracic motion. In relation to this, the ratio of AM to TM (AM/TM) focuses on breathing style (e.g., more AM or more TM during breathing). From the point of statistical analysis, using either AM/(AM + TM) or AM/TM gives the same statistical result. The only statistically significant correlation between AM/(AM + TM) and physical fitness outcomes was with vertical jump height in the 40s age group (r_s_ = 0.178, *p* = 0.032), and the correlation was negligible. These results firmly show that healthy women’s breathing style (from the perspective of breathing movements) is not correlated with physical fitness.

Regarding the different results from different age groups shown in Table 4, it was hard to find a consistent pattern. For example, the relationships were stronger in the young groups than the older groups between AM and jump height (20s: r_s_ = 0.398; 30s: r_s_ = 0.208; 40s: r_s_ = 0.199; 50s: r_s_ = 0.096), between AM and balance (20s: r_s_ = 0.208; 30s: r_s_ = 0.143; 40s: r_s_ = −0.121; 50s: r_s_ = 0.054), and between AM and VO_2_ max (20s: r_s_ = 0.236; 30s: r_s_ = 0.180; 40s: r_s_ = −0.100; 50s: r_s_ = 0.090). On the contrary, the relationship was stronger in the older groups than the younger group for AM and handgrip strength (20s: r_s_ = 0.052; 30s: r_s_ = 0.100; 40s: r_s_ = 0.136; 50s: r_s_ = 0.183). Moreover, the relationships were weak or negligible. Therefore, the magnitude of correlations from different age groups was not obviously different.

AM and AM/(AM + TM) were not correlated with balance. This finding was inconsistent with a previous study that found that larger diaphragm motion during spontaneous breathing was associated with better balance stability [23]. We suggest two reasons for this. First, bias might affect the results of Kocjan’s study, as it enrolled 40 healthy participants, including both men and women aged from 24 to 46, and balance performance was not normalized for body size. Secondly, Kocjan’s study and the present study used different methods. Their study directly targeted the motion of the diaphragm, while ours was indirect. They tested the amplitude of the excursion of the diaphragm using ultrasound, and we tested the displacement of the abdomen and chest using two respiration belts. Technically, using ultrasound to test diaphragm excursion is more accurate. Thus, the use of ultrasound should be considered in future studies.

The current study has some limitations. Firstly, participants’ exercise frequency and intensity were not recorded; this information would be valuable for eliminating confounding factors. Secondly, there were too few male participants, and some of them refused to perform certain physical fitness components (e.g., muscle endurance and cardiorespiratory endurance), so their data were not included in this study. It is necessary to conduct assessments on men in future studies.

## 5. Conclusions

Women’s respiratory movements during spontaneous breathing were not correlated with physical fitness.

Future studies may focus on the relationship between AM and countermovement jump height in young women, with a larger sample size, or using ultrasound instead of respiration belts.

## Figures and Tables

**Table 1:** Interpretation of Spearman rank-order correlation coefficients [53].

Coefficients	Strength
0.00 to 0.20	Negligible
0.21 to 0.40	Weak
0.41 to 0.60	Moderate
0.61 to 0.80	Strong
0.81 to 1.00	Very strong

**Table 2:** Participants’ age, body size, respiratory movements, and physical fitness.

	20s Group	30s Group	40s Group	50s Group
Age	26 (24, 28)	36 (33, 38)	44 (42, 47)	55 (52, 57)
Body height (cm)	162 (158, 165)	161 (157, 165)	162 (159, 165)	160 (158, 164)
Body weight (kg)	56.8 (52.9, 62.5)	58.7 (52.5, 67.0)	61.0 (55.0, 66.8)	61.3 (55.1, 66.5)
BMI (kg/m^2^)	21.6 (19.7, 24.1)	22.5 (20.1, 25.4)	22.5 (21.2, 25.2)	23.7 (21.1, 25.3)
Waist Circumference (cm)	70.2 (67.4, 75.9)	74.3 (68.9, 83.7)	76.3 (71.2, 82.2)	80.0 (73.4, 86.0)
Hip Circumference (cm)	93.3 (91.1, 96.5)	94.0 (88.3, 99.3)	94.3 (90.5, 99.2)	94.9 (90.6, 99.7)
Waist hip ratio (%)	0.75 (0.74, 0.80)	0.80 (0.77, 0.84)	0.80 (0.77, 0.85)	0.84 (0.80, 0.88)
Body fat percentage (%)	26.6 (22.7, 30.1)	27.8 (25.1, 33.5)	29.2 (26.2, 32.3)	30.5 (27.2, 34.3)
Respiration rate (reps/min)	17.3 (15.5, 18.6)	17.2 (15.0, 19.4)	17.2 (13.9, 19.1)	17.0 (14.9, 19.5)
AM (N)	0.97 (0.76, 1.23)	0.84 (0.54, 1.24)	0.87 (0.54, 1.43)	0.99 (0.62, 1.57)
TM (N)	2.20 (1.81, 2.72)	2.31 (1.83, 2.72)	2.49 (1.79, 3.44)	2.20 (1.63, 3.03)
AM + TM (N)	3.25 (2.74, 4.20)	3.10 (2.61, 4.03)	3.53 (2.50, 4.76)	3.05 (2.43, 4.44)
AM/(AM + TM) (%)	30 (22, 44)	25 (18, 38)	29 (18, 38)	32 (23, 39)
Handgrip strength (kg)	23.5 (21.2, 27.1)	25.2 (22.3, 28.2)	26.8 (23.2, 30.2)	24.2 (21.4, 27.0)
Back extension strength (kg)	62.2 (48.5, 68.7)	62.3 (50.3, 73.7)	66.4 (54.9, 78.7)	67.2 (53.7, 76.1)
Vertical jump height (cm)	24.0 (21.4, 27.3)	22.2 (19.0, 25.0)	20.7 (17.6,23.7)	17.8 (14.6, 20.3)
Push-ups (reps/min)	20 (14, 28)	16 (10, 22)	15 (9, 24)	12 (6, 20)
Sit-ups (reps/min)	30 (20, 34)	21 (15, 27)	20 (15, 27)	14 (10, 18)
Balance (sec)	28.3 (12.1, 39.7)	21.2 (11.9, 36.1)	18.8 (10.4, 30.5)	11.5 (6.8, 24.1)
Flexibility (cm/min)	10.5 (3.0, 17.4)	8.5 (1.2, 14.0)	9.7 (4.5, 16.5)	12.3 (5.1, 18.5)
Reaction time (sec)	0.55 (0.52,0.60)	0.57 (0.54, 0.61)	0.59 (0.55, 0.64)	0.62 (0.55, 0.68)
VO_2_max (mL/(kg × min))	44.2 (38.9, 52.3)	40.6 (33.9, 45.2)	36.7 (32.8, 41.3)	32.4 (28.3, 34.3)

Grouping: 20s group included the women aged from 20 to 29; 30s group from 30 to 39; 40s from 40 to 49; and 50s from 50 to 59. BMI = body mass index; AM = abdominal motion; TM = thoracic motion; AM + TM = abdominal motion plus thoracic motion; AM/(AM + TM) = the proportion of abdominal motion to the summation of abdominal and thoracic motion. N = Newton. Please refer to the number of subjects for each parameter in Table 3 and Table 4.

**Table 3:** Correlation between respiratory movements and age, body size, and body fat percentage.

Groups	Physical Performance	Number of Subjects	AM	TM	AM + TM	AM/(AM + TM)
			r_s_	r_s_	r_s_	r_s_
20s group	Age	40	−0.170	0.039	−0.086	−0.196
	Body height	40	−0.116	0.162	0.139	−0.159
	Body weight	40	−0.310	0.106	−0.031	−0.279
	BMI	40	−0.268	0.023	−0.110	−0.217
	Waist circumference	39	−0.181	0.004	−0.059	−0.117
	Hip circumference	39	−0.306	0.055	−0.049	−0.248
	Waist hip ratio	39	−0.049	−0.041	−0.064	0.029
	Body fat percentage	40	−0.264	0.074	−0.050	−0.234
30s group	Age	114	−0.131	0.034	−0.025	−0.121
	Body height	114	−0.078	0.082	0.020	−0.111
	Body weight	114	−0.093	−0.141	−0.142	−0.017
	BMI	114	−0.031	−0.177	−0.142	0.060
	Waist circumference	112	−0.124	−0.162	−0.174	−0.035
	Hip circumference	112	−0.094	−0.116	−0.115	−0.044
	Waist hip ratio	112	−0.084	−0.139	−0.152	0.010
	Body fat percentage	111	−0.062	−0.106	−0.112	0.020
40s group	Age	146	0.122	0.015	0.080	0.122
	Body height	146	0.040	0.109	0.083	0.004
	Body weight	146	−0.016	0.076	0.044	−0.092
	BMI	146	0.006	0.048	0.039	−0.063
	Waist circumference	144	−0.113	−0.062	−0.093	−0.110
	Hip circumference	144	−0.086	−0.021	−0.048	−0.089
	Waist hip ratio	144	−0.089	−0.082	−0.099	−0.086
	Body fat percentage	144	−0.014	0.058	0.031	−0.092
50s group	Age	101	0.024	0.015	0.031	0.001
	Body height	101	−0.137	−0.002	−0.079	−0.185
	Body weight	101	0.000	−0.027	−0.030	−0.010
	BMI	101	0.057	−0.067	−0.023	0.091
	Waist circumference	101	−0.006	−0.034	−0.031	0.004
	Hip circumference	101	−0.034	−0.062	−0.054	−0.021
	Waist hip ratio	101	0.104	0.017	0.051	0.103
	Body fat percentage	100	−0.042	−0.094	−0.091	0.002

AM = abdominal motion; TM = thoracic motion; AM + TM = abdominal motion plus thoracic motion; AM/(AM + TM) = the proportion of abdominal motion to the summation of abdominal and thoracic motion. r_s_ = correlation coefficient of Spearman correlation test.

**Table 4:** Correlation between respiratory movements and physical fitness.

Groups	Physical Performance	Number of Subjects	AM	TM	AM + TM	AM/(AM + TM)
			r_s_	r_s_	r_s_	r_s_
20s group	Handgrip strength	40	0.052	−0.051	0.035	0.123
	Back extension strength	40	0.104	0.213	0.217	−0.018
	Vertical jump height	40	0.398 *	0.065	0.290	0.276
	Number of push-ups	38	0.120	−0.218	−0.203	0.136
	Number of sit-ups	38	−0.030	0.044	−0.032	−0.083
	Balance	40	0.208	−0.068	0.039	0.119
	Flexibility	40	0.031	−0.220	−0.237	0.139
	Reaction time	40	0.074	0.222	0.217	−0.109
	VO_2_max	32	0.236	−0.082	−0.059	0.234
30s group	Handgrip strength	110	0.100	0.124	0.155	0.028
	Back extension strength	113	0.155	0.057	0.157	0.126
	Vertical jump height	113	0.208 *	0.125	0.211 *	0.118
	Number of push-ups	103	0.018	0.121	0.135	−0.057
	Number of sit-ups	103	0.195 *	0.238 *	0.330 **	0.013
	Balance	113	0.143	0.053	0.087	0.115
	Flexibility	110	0.131	−0.021	0.092	0.057
	Reaction time	108	−0.081	−0.168	−0.168	0.031
	VO_2_max	84	0.180	0.187	0.211	0.076
40s group	Handgrip strength	144	0.136	0.089	0.104	0.129
	Back extension strength	140	−0.052	0.068	0.017	−0.070
	Vertical jump height	146	0.199 *	0.129	0.187 *	0.178 *
	Number of push-ups	137	0.102	0.065	0.075	0.088
	Number of sit-ups	134	0.078	0.029	0.061	0.072
	Balance	145	−0.121	0.020	−0.033	−0.089
	Flexibility	145	0.137	0.108	0.140	0.082
	Reaction time	146	0.083	0.039	0.064	0.042
	VO_2_max	113	−0.100	−0.118	−0.098	0.018
50s group	Handgrip strength	100	0.183	0.113	0.167	0.100
	Back extension strength	101	0.104	0.012	0.079	0.108
	Vertical jump height	97	0.096	0.039	0.066	0.111
	Number of push-ups	92	0.168	0.173	0.211 *	0.114
	Number of sit-ups	84	0.075	−0.082	−0.014	0.173
	Balance	101	0.054	0.094	0.091	0.003
	Flexibility	100	−0.094	−0.091	−0.104	−0.065
	Reaction time	99	0.012	−0.007	0.022	−0.025
	VO_2_max	79	0.090	0.022	0.056	0.086

N = number of participants; AM = abdominal motion; TM = thoracic motion; AM + TM = abdominal motion plus thoracic motion; AM/(AM + TM) = the proportion of abdominal motion to the summation of abdominal and thoracic motion. r = correlation coefficient. * *p* < 0.05, ** *p* < 0.01.

## Data Availability

The data that support the findings of this study are available on request from the corresponding author Wenming Liang.
